# Integrated Manufacturing of Suspended and Aligned Nanofibrous Scaffold for Structural Maturation and Synchronous Contraction of HiPSC-Derived Cardiomyocytes

**DOI:** 10.3390/bioengineering10060702

**Published:** 2023-06-09

**Authors:** Lingling Liu, Feng Xu, Hang Jin, Bin Qiu, Jianhui Yang, Wangzihan Zhang, Qiang Gao, Bin Lin, Songyue Chen, Daoheng Sun

**Affiliations:** 1 Sabondong Micron Nano Science and Technology Research Institute, Xiamen University, Xiamen 361102, China; 19920201151452@stu.xmu.edu.cn (L.L.); xu_feng_bp@aliyun.com (F.X.); zwzh2000@163.com (W.Z.); 2 Department of Cardiovascular Surgery, Guangdong Cardiovascular Institute, Guangdong Provincial People’s Hospital, Guangzhou 510080, China; gaoqiang_89@163.com; 3 Guangdong Academy of Medical Sciences, Southern Medical University, Guangzhou 510080, China; 4 Guangdong Beating Origin Regenerative Medicine Co., Ltd., Foshan 528231, China

**Keywords:** hiPSC-derived cardiomyocytes, electrospinning, aligned nanofibers, suspended nanofibers, anisotropic cardiac tissue, maturation

## Abstract

Electrospun nanofiber constructs represent a promising alternative for mimicking the natural extracellular matrix in vitro and have significant potential for cardiac patch applications. While the effect of fiber orientation on the morphological structure of cardiomyocytes has been investigated, fibers only provide contact guidance without accounting for substrate stiffness due to their deposition on rigid substrates (e.g., glass or polystyrene). This paper introduces an in situ fabrication method for suspended and well aligned nanofibrous scaffolds via roller electrospinning, providing an anisotropic microenvironment with reduced stiffness for cardiac tissue engineering. A fiber surface modification strategy, utilizing oxygen plasma treatment combined with sodium dodecyl sulfate solution, was proposed to maintain the hydrophilicity of polycaprolactone (PCL) fibers, promoting cellular adhesion. Human-induced pluripotent stem cell (hiPSC)-derived cardiomyocytes (CMs), cultured on aligned fibers, exhibited an elongated morphology with extension along the fiber axis. In comparison to Petri dishes and suspended random fiber scaffolds, hiPSC-CMs on suspended aligned fiber scaffolds demonstrated enhanced sarcomere organization, spontaneous synchronous contraction, and gene expression indicative of maturation. This work demonstrates the suspended and aligned nano-fibrous scaffold provides a more realistic biomimetic environment for hiPSC-CMs, which promoted further research on the inducing effect of fiber scaffolds on hiPSC-CMs microstructure and gene-level expression.

## 1. Introduction

Cardiac failure is a major global health concern, with myocardial infarction (MI) being a leading cause of death associated with cardiovascular disease [1]. Building engineered cardiac tissue models and cardiac patches in vitro is one important route to study cardiovascular disease or regenerate cardiac tissue [2,3,4]. Human-derived induced pluripotent stem cells (hiPSCs) possess a nearly unlimited capacity for self-renewal and can theoretically differentiate into an unlimited number of cardiomyocytes (CMs) [5,6], providing a promising platform for cardiac patch applications [7,8]. However, achieving phenotypic maturation of hiPSC-CMs comparable to adult human cardiomyocytes remains a challenge [9]. HiPSC-CMs cultured on Petri dishes tend to be small, mononucleated, and rounded with disorganized and shorter sarcomeres [10]. Although hiPSC-CMs become progressively mature in long-term culture (a full year) [11], the time cost limits their practical application. Thus, there is an urgent need for effective scaffolds to enhance hiPSC-CM maturation.

Anisotropy is an important factor in achieving mature phenotypes in the morphology and structure of hiPSC-CMs [12]. The heart is a complex three-dimensional anisotropic structure with a helically organized myocardial band and slight interlayer arrangement shift from the endocardium to the epicardium according to the Torrent-Guasp model [13,14,15]. Anisotropic polymer scaffolds can provide contact guidance for cellular reorganization and functional modulation [16,17,18]. The current main approaches to fabricating anisotropic tissue engineering scaffolds include micro-nano-groove substrate [19,20,21,22] and aligned fibrous scaffolds [23,24,25,26] based on lithography (or soft lithography), as well as electrospinning. Electrospinning has attracted significant attention for the in vitro construction of anisotropic microstructures that resemble the natural ECM [27,28]. Evidence shows the CMs cultured on the anisotropic scaffolds stretched in the structural direction to obtain a higher aspect ratio, higher sarcomere length, faster Ca^2+^ propagation speed, and synchronous contraction rate, showing more mature electrophysiological properties than two-dimensional flat plates [29,30]. Anisotropy influences electrical signal propagation by promoting the polarization of extracellular gap junctions to the longitudinal ends of the cardiomyocytes [31,32,33]. 

Although many scholars have reported the induction effect of aligned fiber scaffolds on myocardial cells, currently the aligned fibers are firstly cut into certain shapes and then transferred to glass substrates [34,35]. When fibers are fixed around the substrate, cells perceive the stiffness of the plastic substrate (~2 GPa), and the fiber orientation structure only provides contact guidance [36,37]. However, when fibers are suspended on a support frame, cells perceive the stiffness of the support frame connecting the two ends of the aligned fibers. A cell at the center of the aligned fibers is only hundreds of microns from the rigid support, whereas for suspended fibers, the rigid support is millimeters away. By adjusting the material and structure of the support frame, it is possible to prepare a scaffold with stiffness that matches the myocardial tissue modulus. These mechanical nuances may significantly impact the contractile behavior of myocardial cells.

Here, we propose an in situ method for fabricating suspended and well aligned polycaprolactone (PCL) nanofibrous scaffolds via roller electrospinning. Specifically, aligned fibers suspended on PDMS microbeams do not require transfer and provide a three-dimensional anisotropic environment with reduced stiffness for hiPSC-CMs. HiPSC-CMs are only constrained by the fiber direction and can freely extend in the other two dimensions from a microscopic perspective. Surface modification with oxygen plasma combined with sodium dodecyl sulfate (SDS) solution was employed to maintain fiber hydrophilicity, promoting cell–fiber adhesion and uniform distribution. Compared to random fibers and Petri dishes, hiPSC-CMs cultured on suspended and aligned fibrous scaffolds exhibited rod-like morphology and ordered sarcomere organization. The MYH7/MYH6 protein concentration ratio for aligned fibers was three-fold higher, indicating enhanced structural morphology and gene expression maturation of hiPSC-CMs.

## 2. Materials and Methods

### 2.1. Fabrication of Aligned Scaffolds

Polycaprolactone (PCL) (Mn = 80,000, Sigma Aldrich, Darmstadt, DE) was dissolved in 20% *w*/*w* in glacial acetic acid (99.7%, Shanghai Trial, Shanghai, China). The mixture was loaded into a 5 mL syringe and delivered through a 25-gauge blunt-tip needle at a flow rate of 0.5 mL/h using a syringe pump (D300401, Harvard Apparatus, Holliston, MA, USA). Electrospinning was realized by a high-voltage power supply (DW-P503-1ACD1, Dongwen High Voltage, Tianjin, China) onto a grounded stationary collector placed at a distance of 12 cm from the tip of the syringe. The collector was kept static for 10 min to obtain randomly distributed fibers, with a power supply of 8.5 kV. For the aligned fibers, the collector was set at a rotating speed of 1500 rpm for 10 min.

**Figure 1:**
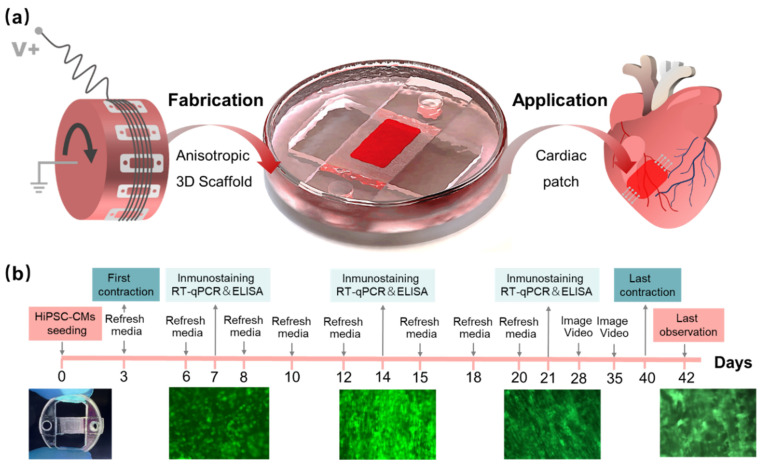
Preparation of aligned fibers and process of hiPSC-CMs culture. (**a**) Aligned fiber scaffold prepared by electrospinning in roller, suspension culture of hiPSC-CMs, and final application for the cardiac patch; (**b**) the whole culture process of hiPSC-CMs on the scaffold.

The supporting substrates were fabricated by soft lithography, with the molds printed by stereolithography (S240, BMF Precision Tech Inc., Chongqing, China). PDMS (10:1, Sylgard184, Dow Corning) was cured at 80 °C for 30 min. The flexible PDMS films with microstructure were attached tightly to the roller (Figure 1a). Electrospinning was performed according to the above process parameters, and the PDMS film with aligned fiber was fixed on a PMMA circular frame. The whole circular device was placed in a twelve-well plate to ensure fiber suspension in media (Figure 1a), and the resultant culturing states are shown in Figure 1b.

### 2.2. Characterization of the Nanofibers

The morphology of the fibers was observed with scanning electron microscope (SEM) (JSM-IT500A, JEOL, Tokyo, Japan). SEM images were processed in ImageJ (NIH, Bethesda, USA) to assess the orientation of the fibers. Oval projection of the Fast Fourier Transform (FFT) of the images were performed, followed by a radial summation of the pixel intensities for each angle between 0° and 180° (because FFT data are symmetric). The alignment degree is reflected by the data distribution, where the peak height and shape indicate the principal angle of orientation. The mean fiber diameter and the standard deviation were calculated by measuring 100 points on both random and aligned samples. Finally, the stiffness of the fibers was measured with a tensile strain assay using an electromechanical universal testing machine (E43, Meters Industrial Systems, Hopkins, MN, USA) with tensile speed of 50 mm/min (*n* = 6).

### 2.3. Modification and Characterization of Hydrophilic Fibers

The surface of PCL fibers was firstly treated with oxygen plasma (PLUTO-T, Peiyuan Instrument, Shanghai, China) to improve hydrophilicity. Oxygen entered the chamber at an intake rate of 150 mL/min and was processed at a power intensity of 30 W for 60 s. Then, the sample was treated with 0.5% SDS solution (PBS as solvent) and then dried naturally at room temperature. The change in contact angle for untreated PCL fibers (Untreated), oxygen plasma-treated PCL fibers (Plasma), and oxygen plasma and SDS PCL fibers (Plasma + SDS) was recorded with a contact angle meter (JC2000D5W, Zhongchen digital technology equipment, Shanghai, China). X-ray photoelectron spectroscopy (XPS) was used to characterize the functional groups of the fiber surface. The binding energy range was 1188.68~1207.68 eV, and the pass energy was 30 eV.

### 2.4. Generation of HiPSC-Derived Cardiomyocytes

The mouse HL-1 cells were purchased from iCell Bioscience. HiPSC (Beijing Cellapy Biological Technology Co. Ltd., Beijing, China) were cultured on matrigel-coated (0.15%, Corning, 356234) six-well plates in StemFlexTM medium (Gibco, A3349401) at 37 °C in 5% CO_2_. The hiPSCs were labeled with green fluorescent by introducing an EGFP expressional element in an AAVS1 locus. HiPSC-CMs were cultured according to a previous publication [38]. Briefly, hiPSCs were treated with small molecule CHIR99021 (MedChemExpress, HY-10182, final concentration 10 μM) in the RPMI-BSA medium [RPMI 1640 Medium (Gibco, C11875500BT), supplemented with 213 μg/mL AA2P (l-ascorbic acid 2-phosphate magnesium) (Sigma, 49752) and 0.1% bovine serum albumin (BSA) (Sangon Biotech, A600332-0100)] for 24 h, and then they were incubated with RPMI-BSA medium for 48 h. On differentiation day 4, cells were treated with the small molecule IWP2 (MedChemExpress, HY-13912 final concentration 5 μM) in the RPMI-BSA medium. After 48 h, the media were changed to RPMI-BSA medium. Then, RPMI 1640 Medium supplemented with 3% KnockOut Serum Replacement (Gibco, 10828-028) was used to culture the cardiomyocytes in the following experiments. For purification, DMEM Medium (No Glucose) (Gibco, 11966-025) supplemented with Sodium DL-lactate (Sigma, L4263, final concentration 4 mM) was used. The purification process lasted no longer than 9 days, and the medium was changed every 2~3 days. The purity of cardiomyocytes after purification is about 92.91% in our study. The results and method are presented in Figure S1.

### 2.5. Seeding and Culture of the HiPSC-CMs

Before cell seeding, all devices were sterilized by γ ray irradiation (Irradiation dose is 30 KGy.) for 5 min and coated with porcine gelatin (Sigma, V900863) at 0.15% *w*/*v* in distilled PBS for 1 h at 37 °C. Firstly, cardiomyocytes were digested with 0.05% trypsin. Approximately 20 µL of the cell suspension (∼50,000 cells/well) were seeded on a fibrous scaffold. All devices were incubated at 37 °C for approximately 4 h to allow cell attachment. Then, the devices were cultured with RPMI 1640 Medium supplemented with 3% KnockOut Serum Replacement in a 37 °C, 5% CO_2_ incubator with media being changed every 2~3 days for the duration of the experiment (42 days).

### 2.6. Cell Proliferation Analysis

The proliferation of mouse HL-1 cells was tested, seeding on hydrophilic fibers without gelatin coating, hydrophilic fibers with gelatin coating, hydrophobic fibers without gelatin coating, and hydrophobic fibers with gelatin, respectively. Cell proliferation was analyzed using Microplate Reader (K3 Touch, Thermo Fisher Scientific, Somerset, NJ, USA) and an enhanced CCK-8 kit (C0043, Beyotime, Shanghai, China). Different groups of cell-seeding scaffolds were washed three times with PBS. Then, 1500 µL DMEM medium and 150 µL CCK-8 were added to each well of 24-well plates, and incubated for 3 h. Finally, the solutions were transferred to a 96-well plate (200 µL per well) to read the OD values at a wavelength of 450 nm.

### 2.7. HiPSC-CMs Contraction Analysis

Measurement of cell contraction frequency using optical methods was performed for automatic analysis of fluorescence images. Firstly, a video of the spontaneous contraction of hiPSC-CMs was captured in real-time using an inverted fluorescence microscope. The video was imported into ImageJ software and divided into a series of single frame images (.tiff) to generate an image stack. Select the middle area of the rectangular box to detect the fluorescence intensity change in the video with the increase in frame number (Image -> Stacks -> Plot Z-axis Profile), and obtain a waveform of the green fluorescence intensity change with the increase in frame number. Furthermore, the average number of frames required for each jump is obtained by measuring the distance between each wave peak and taking the average value. Finally, obtain the contraction frequency of myocardial cells according to the formula: Beat Rate (beats/minute) = 60×(average frames required per beat/frames per second of video).

### 2.8. Immunostaining, Quantification of Cell Alignment, and Cx-43 Expression

For immunofluorescence staining, cardiomyocytes were fixed with 4% paraformaldehyde for 20 min, followed by permeabilization with 0.25% Triton-X 100 (Sigma-Aldrich) in Phosphate buffered saline (PBS) for 20 min at room temperature. After incubating in the blocking buffer (10% goat serum in PBS), cells were stained with primary antibodies (Monoclonal Anti-a-Actinin (Sarcomeric), sigma, A7811; Anti-Connexin-43 antibody, sigma, c6219) at 4 °C overnight. Cells were washed three times with PBS containing 0.1% Triton X-100, then fluorescent secondary antibodies (Alexa Fluor555 goat anti-rabbit, Invitrogen, Az1428; Invitrogen, A21127) were added for 60 min in the dark at 37 °C. Nuclei were labeled with Hoechst 33342 (Beyotime, C1022) for 10 min. The samples were washed with PBS at least 3 times between each step and maintained at 4 °C in PBS until image acquisition.

Imaging of the cultured cells was performed on an inverted microscope (MF-52N, Mshot, Guangzhou, China) with the same acquisition parameters for all the samples. ImageJ was used to evaluate all fixed and stained cells for differences in nucleus alignment, aspect ratio, sarcomere length, sarcomere alignment direction, and area coverage of connexin-43 (CX-43) proteins. To quantify cell alignment, we used the plugin Orientation J to obtain the orientation and isotropy properties of the images based on the evaluation of the gradient structure tensor. Import fluorescent images of the CX-43 protein into ImageJ software, and the editing program will automatically read the fluorescent areas of multiple images, and then it will export the data to Origin for processing and analysis.

### 2.9. Real-Time Quantitative PCR

The total RNA of hiPSC-CMs was extracted using the EZ-10 Total RNA Mini-Preps Kit (BBI, B618583-0100). The Hifair^®^ III 1st Strand cDNA Synthesis Kit (YEASEN, 11139ES60) was used to convert 500 ng of mRNA to cDNA following the manufacturer’s directions. Quantitative PCR was performed using a Light Cycler 480 (Roche) with TB^®^ Green Fast qPCR Mix (TaKaRa, RR430A). Running conditions were 95 °C 10 min followed by 40 cycles of 95 °C for 15 s and 60 °C for 60 s. Reactions were performed in triplicate, and expression levels were calculated using the CT comparative method (2^−ΔΔCT^). As shown in Table 1, GAPDH was used as a housekeeping gene, and the quantitative PCR primers designed from qPrimerDepot [39] are as follows (from 5′ to 3′).

### 2.10. Enzyme-Linked Immunosorbent Assay (ELISA)

We used the enzyme-linked immunosorbent assay (ELISA) method to detect the protein concentration of MYH7 and MYH6, and then we calculated the MYH7/MYH6 ratio. The assay kits were purchased from ELK Biotechnology (ELK4362). HiPSC-CMs supernatant at 1000× *g* rpm centrifuge for 20 min to remove impurities and cell debris. Firstly, balance the assay kit and supernatant at room temperature. Add 100 μL supernatant diluent buffer to each well of the pre-coated microplate, and incubate at 37 °C for 80 min. Next, discard the liquid from the pre-coated microplate and add 200 μL wash buffer to each well. Wash 3 times. After drying, add 100 μL biotinylated antibody dilute to each well, and incubate at 37 °C for 50 min. After that, discard the liquid from the pre-coated microplate, and add 200 μL wash buffer to each well. Wash 3 times. After drying, add 100 μL HRP diluent to each well, and incubate at 37 °C for 50 min. Then, discard the liquid from the pre-coated microplate, and add 200 μL wash buffer to each well. Wash 5 times. After drying, add 90 μL TMB substrate solution to each well, and then incubate at 37 °C for 20 min. Finally, add 50 μL stop reagent to each well, measure the absorbance (OD value) using an enzyme-linked immunosorbent assay at a wavelength of 450 nm, and calculate the sample concentration by plotting a standard curve.

### 2.11. Statistical Analysis

Quantitative data on CMs orientation, aspect ratio, sarcomere length, sarcomere alignment direction, and coverage area of CX-43 protein were analyzed using ImageJ software, resulting from at least 5 images (cell number > 200) at 3 independent locations for each sample and expressed as means ± standard deviations. Statistical differences were obtained by ANOVA followed by a post hoc Tukey’s test for significant differences. A significance level of 0.05 was applied to determine significant differences.

## 3. Results and Discussion

### 3.1. Aligned Fibrous Scaffolds

**Figure 2:**
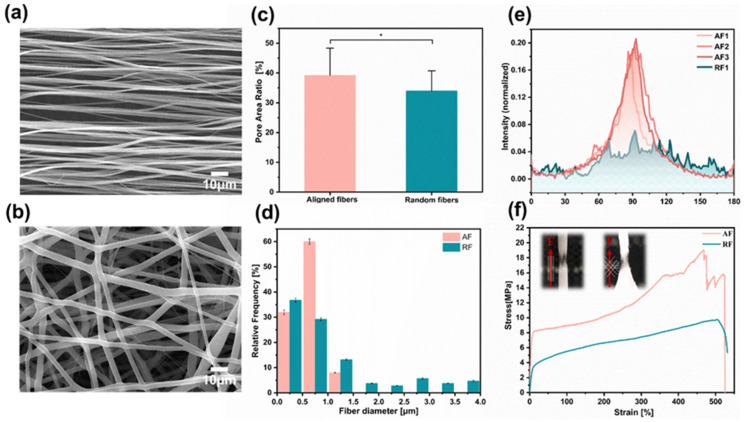
Electrospun fibers characterization. (**a**,**b**) SEM images show the morphology of the aligned and random fibers, respectively. (**c**) Mean pore area ratio of the aligned and random fibers. (**d**) Mean nanofiber diameter for each of the conformations. (**e**) FFT analysis of the nanofiber orientation based on the SEM images. (**f**) Characteristic stress–strain curves were obtained for the tensile mechanical assay of each sample type. Scale bar is 10 µm in (**a**,**b**). Data in (**c**,**d**) are expressed as means ± standard deviations (*n* = 12) with * *p* < 0.05 (evaluated with student’s *t*-test).

Roller electrospinning and far-field electrospinning are the most effective methods to manufacture aligned and random fiber scaffolds [40], which can acquire superior morphology and mechanical properties of fibers, as shown in SEM micrographs (Figure 2a,b). Fiber density is an important factor that affects cell seeding efficiency. Pre-experiments show that the lower-density fibers (electrospinning for 5 min) tended to be damaged during cell seeding, while for the higher-density fibers (electrospinning for 15 min), there was not enough space for cells to extend freely. Therefore, a moderate fiber density is chosen by electrospinning for 10 min. Here, we counted the porosity of fibers by image processing software and found that the porosity of aligned fibers was 36.11% ± 9.07% and that of random fibers was 33.98% ± 6.73%, regarded as moderate fiber density, by spinning for 10 min (Figure 2c). The average diameter of Aligned Fibers (AF) was 0.56 ± 0.13 μm, and, for Random Fibers (RF), it was 0.80 ± 0.33 μm (Figure 2d). The difference in fiber orientation was quantified by FFT analysis and radial summation of the elliptical profiles of the SEM images. A clear peak at 90° is observed for aligned fibers, while for randomly distributed fibers, a random distribution at different angles is seen (Figure 2e). The tensile modulus of random nanofibers is isotropic with a mean value of 62.77 ± 5.44 MPa. In comparison, the mean elastic modulus of aligned fibers stretched parallel to the fiber direction is 143.54 ± 44.97 MPa (Figure 2f and Figure S2).

By using three-dimensional printing to prepare molds, we can customize PDMS frames. We utilize the flexibility of PDMS and the cylindrical drum to manufacture aligned fiber scaffolds in one step, avoiding deformation and damage caused by fiber transfer and cutting. Specifically, the PDMS framework (thickness is 300 μm) with a hollow rectangle structure is attached to the drum, and tape is used as a mask. A drum with a diameter of 20 cm can rapidly prepare 24 suspended fiber scaffolds at once. We speculate that, when fibers are directly fixed on a rigid substrate, hiPSC-CMs need to conquer the high stiffness of the fibers personally during contraction. However, when the fibers are suspended in the PDMS framework, hiPSC-CMs actually pull the PDMS micro beams at both ends of the fixed fibers during contraction, which may be beneficial for the spontaneous contraction of hiPSC-CMs due to lower stiffness. For the convenience of operation and observation, a PDMS frame with fibers was fixed on a 2 mm thick PMMA circular support, which also facilitates our subsequent electrical stimulation experiments. Overall, the drum electrospinning process can meet the demand for robust, reproducible, cost-effective, and engineered mass production of nanopatterned myocardial tissue scaffolds. It is a single-step procedure performed with commonly used equipment (such as pumps, or a high-voltage source) and well-performed biocompatible biomaterials.

### 3.2. Hydrophilic Fibrous Scaffolds

**Figure 3:**
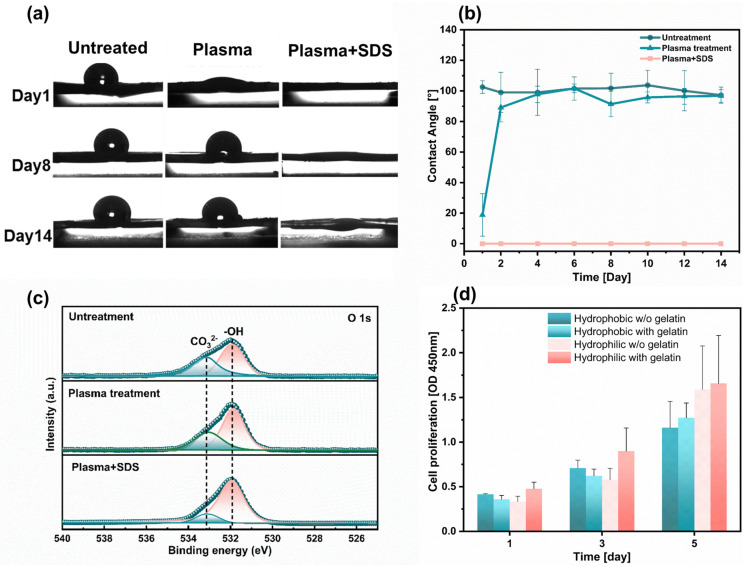
Fabrication and characterizations of the hydrophilic fiber. (**a**) Water contact angle of fiber surface after different treatment (*n* = 3). (**b**) Changes in contact angle of fibers with increasing days (*n* = 3). (**c**) XPS patterns of O 1 s revealed different peak areas in the -OH hydrophilic group for the three groups of samples. (**d**) Cell proliferation of HL-1 cells seeded on aligned fibers with different surface treatments (*n* = 6).

Hydrophobicity is an inherent attribute of the PCL polymer. When cells are seeded on PCL fiber scaffolds in droplets, cell penetration into the interior of the nanofiber is restricted [23]. For common polymer materials, the value of contact angle is used to quantitatively determine whether they are hydrophilic. When the value of the contact angle is ≥90°, it is defined as hydrophobic. When we add a certain volume of water droplets to the surface of fibers, different morphologies will be formed. As shown in Figure 3a, the water droplets on the fiber surface treated with Plsama + SDS have completely penetrated compared to the obvious spherical droplets on the untreated fiber surface. By measuring the angle between the tangent line of the water droplets and the intersection line of the fibers determines the contact angle of each group of samples (Figure 3b). The average contact angle of untreated PCL fibers tested within 14 days is 100.60° ± 2.16°, and the fibers treated by plasma regained their hydrophobic after 2 days. The fibers’ surface properties were analyzed by XPS analysis on the 14th day after hydrophilic treatment. The peak area of the −OH hydrophilic group located at position 531.9 eV gradually increases from Untreated, Plasma to Plasma + SDS (Figure 3c).

HL-1 cells were cultured on hydrophilic fibers without gelatin coating, hydrophilic fibers with gelatin coating, hydrophobic fibers without gelatin coating, and hydrophobic fibers with gelatin, respectively. The proliferation rate of the HL-1 cells seeded on the hydrophilic fibers was 1.4 folds as high as that of the hydrophobic fibers during the 5-day culture period, thereby confirming that the addition of thin fibers was conducive to cell proliferation. We further found that more HL-1 cells adhere to the homophilic fibers coated-gelatin (Figure 3d), which is consistent with numerous studies on the effect of incubation in protein solution of the scaffolds before cell seeding [34]. When the fibers are in a hydrophobic state, the gelatin solution cannot penetrate into the interior of the scaffold to coat the surface of the fibers. Failure to coat the surface of the Petri dish or fiber scaffold with gelatin will reduce the adhesion between cells and fibers. However, most histiocytes cannot survive at all when they are dissociated and suspended in the liquid. As adherent cells, cardiac myocytes need to adhere to solid or semi-solid surfaces with appropriate positive charges to grow normally [41]. Hydrophilic fiber surface promotes gelatin coating. Gelatin provides more RGD on the fibrous scaffold surface, which is recognized by the integral proteins. The cells and fibers form focal adhesion (FA) connections through receptor and ligand binding [42,43,44].

### 3.3. Myocardial Tissue with Highly Anisotropic

**Figure 4:**
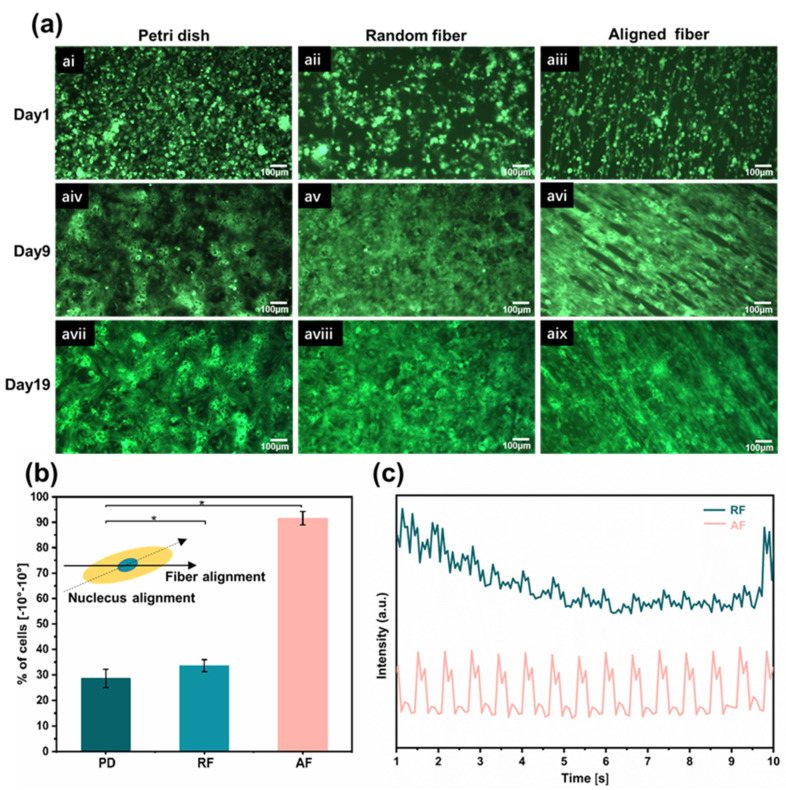
Myocardial tissue with high anisotropy. (**a**) With increasing the culture time, changes in morphological structure of GFP-hiPSC-CMs on Petri dishes, random fibers, and aligned fibers, respectively. (**b**) Cell orientation was assessed by the angle between the long axis of the nucleus and the orientation of the aligned fibers. Results represent the percentage of cells orientation between −10° and 10°. (**c**) The frequency of spontaneous synchronous contraction of CMs on aligned and random fibers. Data in (**b**) is expressed as means ± standard deviations (*n* = 6) with * p < 0.05 (evaluated with student’s *t*-test).

To facilitate real-time observation of cell morphology and contraction, hiPSC-CMs marked with green fluorescent protein (GFP) were seeded on 2D Petri dishes (PD), random fiber scaffolds (RF), and aligned fiber scaffolds (AF). AF and RF were suspended in media, and the cardiomyocytes were constrained by the fiber orientation only in one dimension and were free to migrate in the other two dimensions [45]. The growth of the three groups of samples was recorded separately during culture. On the first day of seeding, the cells in each group were round and independently distributed. As the number of culture days increased, the distribution of CMs contractile protein was isotropic in the Petri dishes and random fibers, showing no preferential orientation angles and forming intercellular clusters. In contrast, the cells elongated significantly in the aligned fiber scaffold, acquiring a shuttle shape that performed obvious anisotropic characteristics. An orderly intercellular arrangement into a patchwork of tissue was observed, as shown in Figure 4a. The cell arrangement behavior was quantified by measuring the angle between the aligned direction of the fibers and the direction of the long axis of the nucleus. We defined that an angle within ±10° could be considered as an orientation of the cells, and the results showed that the orientation of the cells on the aligned fiber scaffold was 91.6%, which was three times higher than on the Petri dish and random fibers (Figure 4b). CMs contracted spontaneously on the third day after seeding, and the frequency of spontaneous synchronous contraction of CMs on aligned fibers were higher than those on random fibers (Figure 4c, Videos S1 and S2). Prolonged incubation of cells on aligned fibers was performed, where spontaneous contraction of the myocardial tissue was recorded even on day 38 and viability on day 42 (Figure S3).

### 3.4. Immunofluorescence Staining of Cardiomyocytes

**Figure 5:**
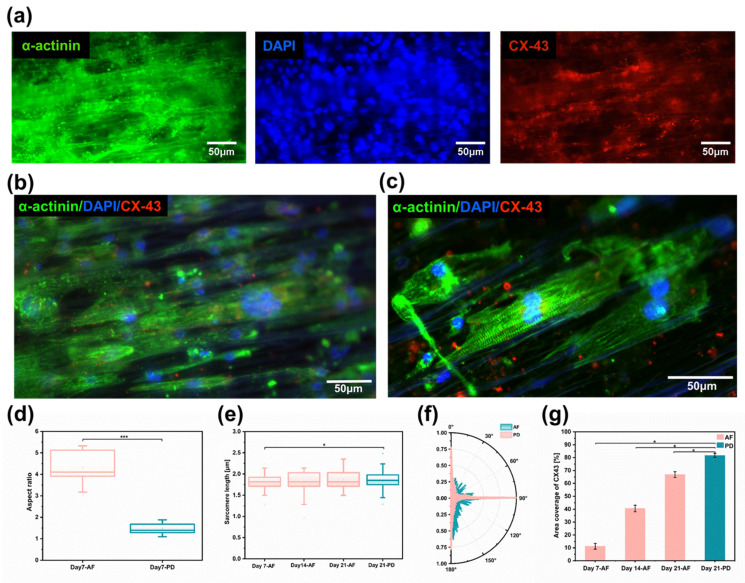
Myocardial tissue with high anisotropy. (**a**) Images of immunofluorescence staining for cardiomyocyte protein α-actinin (green), nucleus (blue), and gap junction protein (red). (**b**) Cardiomyocytes extension and growth along the fiber direction on aligned fibers. (**c**) Cardiomyocytes with special sarcomere arrangement; (**d**) Cardiomyocyte length-to-width ratio (*n* = 6) on aligned fibers versus two-dimensional Petri dishes on day 7. (**e**) Cardiomyocytes sarcomere length on aligned fiber scaffolds (*n* = 6) on the 7th, 14th, and 21st days. (**f**) Sarcomere arrangement direction of cardiomyocytes on aligned fiber scaffolds on day 7 (*n* = 6). (**g**) Area coverage of CX-43 protein on aligned fiber scaffolds on days 7, 14, and 21 (*n* = 6). Data in (**d**,**e**,**g**) are expressed as means ± standard deviations with * p < 0.05 and *** p < 0.001 (evaluated with student’s *t*-test).

Immunofluorescence staining results showed that CM on aligned fiber scaffolds on day 7 expressed a more mature phenotype by analyzing the expression of the cardiac markers sarcomeric α-actinin (α-actinin), nucleus (DAPI), and gap junctional protein (CX43) (Figure 5a and Figure S4)). Adult myocardial cells exhibit an elongated anisotropic rod-shaped shape with an aspect ratio ranging from 7 to 9.5. By counting the aspect ratios of individual cells on the synthetic images (Figure 5b), it was found that, under the induction of aligned fibers, the morphology of hiPSC-CMs exhibited an anisotropic shuttle-like shape by the seventh day of culture, with a maximum aspect ratio of 7 and an average aspect ratio of 4.5, approximately three-fold that of the Petri dish (Figure 5d). The isotropic flat substrate provided no contact guidance for hiPSC-CMs, which resulted in hiPSC-CMs having a spherical morphology and lower aspect ratio. Sarcomeres are a crucial marker of vital CM contractility and maturation, and CMs on aligned fiber scaffolds expressed well organized sarcomere α-actin [46]. Sarcomeres were arranged orderly along the fiber direction (Figure 5c). Statistical analysis of the sarcomere length of cardiomyocytes after the 7th day, the 14th day, and the 21st day of culture revealed that the sarcomere length gradually increased with the increase in culture time, and the sarcomere length of aligned fibers was 1.88~2.1μm at day 21 (Figure 5e). The sarcomere alignment of aligned fibers was concentrated at 90° ± 10°, and the sarcomere alignment of Petri dishes was evenly distributed within 0~180° (Figure 5f). Sarcomere alignment along a major axis was encouraged by anisotropic scaffolds, which allows unidirectional, as well as forceful contraction, as compared to randomly oriented sarcomeres. CX43 is a crucial gap junction protein that allows the passage of ions and solutes between cells, which contributes to intercellular communication and the regulation of electrical and mechanical connections between adjacent cardiomyocytes. CMs on aligned fiber scaffolds showed an increasing trend of CX-43 protein with increasing culture time (Figure 5g). The suspended aligned fiber scaffold is a three-dimensional porous structure (as shown in Figure S5), and the CMs are distributed in a three-dimensional space. Additionally, the CX-43 fluorescence image is a two-dimensional imaging, which can only focus on a certain two-dimensional plane. While adding the same number of cells on a two-dimensional culture dish, the cell density on the two-dimensional substrates would be much higher than that on the three-dimensional scaffolds, resulting in the area coverage of Cx-43 on AF-21 being less than PD-21.

### 3.5. Cardiomyocyte Gene and Protein Expression

**Figure 6:**
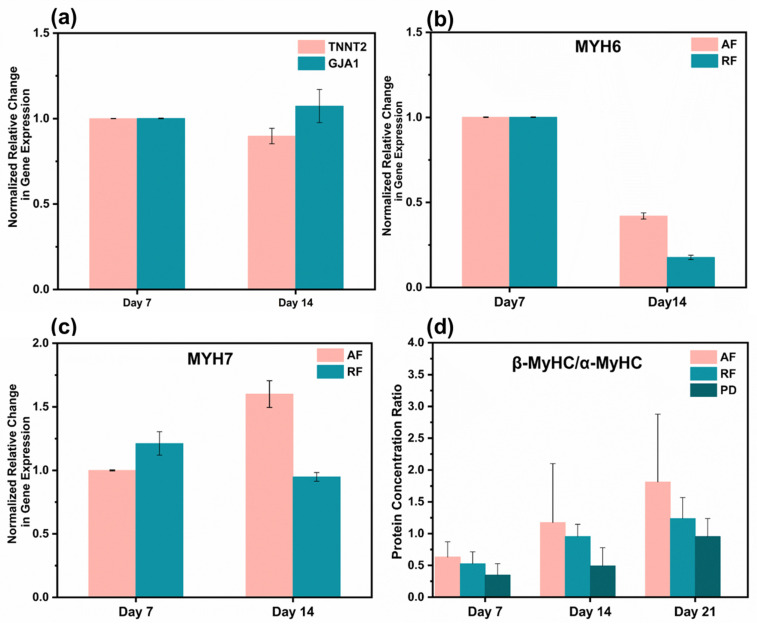
Gene and protein expression analysis. (**a**) Cardiac troponin T (encoded by the TNNT2 gene) and Connexin-43 (encoded by the GJA1 gene). (**b**) Myosin heavy chain alpha isoforms (encoded by the MYH6 gene). (**c**) Myosin heavy chain beta isoforms (encoded by the MYH7 gene). (**d**) Protein concentration ratios of β-MyHC/α-MyHC of cardiomyocytes on aligned fibers, random fibers, and Petri dishes on days 7, 14, and 21. Gene expression values are computed as fold changes using the ∆Ct method.

We further consider the effect of nanofibers on driving cell maturation by analyzing the transcriptional expression of several critical cardiac markers. The TNNT2 gene was quantified to assess the contractility of cardiomyocytes, which encodes the protein of cardiac troponin T (cTnT), located on the thin filaments of the transverse muscle and regulates muscle contraction through changes in intracellular calcium ion concentration [47]. Functional gap junctions and intercellular coupling in myocardial tissue were quantified by analyzing the GJA1 gene, which encodes the CX-43 protein [48]. As the number of cultivation days increased, there was no significant difference in the expression of TNNT2 and GJA1 genes on aligned fibers (Figure 6a), indicating that the fibers are not impairing cardiac survival. In addition, due to the purity of cardiomyocytes after purification being about 92.91%, 7.08% of non-CMs were cultured on the fibrous scaffold. The result indicated that the non-CMs do not proliferate on the fibrous scaffold once the expression of TNNT2 on day 7 was similar to the one observed on day 14. The MYH6 and MYH7 genes, which encode the α- myosin heavy chain (α-MyHC) and β- myosin heavy chain (β-MyHC), respectively, were evaluated. As a vital component of the thick constituent filaments (thick filaments), the β-subunit is associated with a more adult-like phenotype, and the α-subunit tends to be higher in fetal expression [49]. We found that the expression of the MYH6 gene on aligned fibers and random fibers gradually decreased with increasing days of culture (Figure 6b). In contrast, the expression of MYH7 gene increased gradually, with aligned fibers being higher than that of random fibers (Figure 6c). This result is consistent with the gradual developmental maturation of cardiomyocytes in vivo [6]. We quantified the β-MyHC/α-MyHC ratio by ELISA, which generally increases with the developmental stage [50]. The protein concentration ratios of aligned fibers on day 21 were 2.95 times higher than those of day 7 and at all periods were higher than those of random fibers and Petri dishes. Most importantly, the β-MyHC/α-MyHC ratios of aligned fibers were two times higher than those of Petri dishes on day 21 (Figure 6d). The conjectured reason is the highly aligned arrangement of cells on aligned fibers allows gap junction proteins to be distributed at the longitudinal ends of the CM [51], facilitating the synchronous propagation of unidirectional signals and the increased conduction velocity, which requires more energy.

Furthermore, as it has been reported in the literature that myosin (encoded by the MYH gene) is a hexameric enzyme that hydrolyzes ATP [52], spontaneous cell contraction requires a large amount of myosin to provide energy, leading to the upregulation of the MYH7 gene, which correlates with the result that anisotropic structural scaffolds will affect hiPSC-CM electrochemical coupling and mitochondrial morphology [53]. On the other hand, cells contracted along the direction of aligned fibers, and myosin alignment, were highly ordered in the direction of myosin, which allowed the contraction–diastole process to generate forces in the same primary direction, and this enhanced the overall contractile force of myocardial tissue [54]. Spontaneous contraction of CMs on aligned fibers can be considered as passive mechanical training. As shown in some published articles, periodic mechanical stretching increases the MYH7/MYH6 ratio, indicating developmental CM maturation [55].

## 4. Conclusions

In this study, we achieved in situ preparation of aligned PCL fibers by roller electrospinning, which can allows efficient batch fabrication. A new strategy using oxygen plasma with SDS surface modification was performed to keep the fibers hydrophilic for a longer time and to ensure uniform distribution of cells during seeding. Culturing of hiPSC-CMs demonstrates that the cells on aligned fiber scaffolds are more mature in morphology and function compared with random fibers and Petri dishes, which could further be improved by introducing electrical stimulation to the system. It is expected that aligned PCL fiber scaffolds cultured with hiPSC-CMs have great potential to be applied for cardiac patches for genuinely personalized and precise treatment of heart diseases.

## Figures and Tables

**Table 1:** The primers used in the real-time quantitative PCR assay.

Gene	Forward (5′→3′)	Reverse (5′→3′)
*GAPDH*	CGCTCTCTGCTCCTCCTGTT	CCATGGTGTCTGAGCGATGT
*TNNT2*	AGAGGAGGACTGGAGAGAGG	GTCCACTCTCTCTCCATCGG
*GJA1*	AGCAGTCTGCCTTTCGTTGT	TCTGCTTCAAGTGCATGTCC
*MYH6*	CCGATAGTGCCTTTGACGTG	TGACATACTCGTTGCCCACT
*MYH7*	TGGGCTTCACTTCAGAGGAG	TGACGTACTCATTGCCCACT

## Data Availability

We couldn’t provide details of the research data due to privacy restrictions.

## Supplementary Materials

The following supporting information can be downloaded at: https://www.mdpi.com/article/10.3390/bioengineering10060702/s1, Figure S1: The purification efficiency of hiPSC-CM in this study; Figure S2: The stress-strain curve of PCL Aligned Fibers (AF) and Random Fibers (RF); Figure S3: HiPSC-CMs marked GFP cultured on aligned fibers for 42 days; Figure S4: Immunofluorescence staining of hiPSC-CMs cultured on aligned fibers for 7 days. (Green:α-actinin; Blue: DAPI; Red:CX-43); Figure S5: SEM image of ordered fiber scaffold cross-section. Video S1: Spontaneous Contraction of HiPSC-CMs on Aligned Fibers; Video S2: Spontaneous Contraction of HiPSC-CMs on Random Fibers.
