# Supplementary material for: Integrated Manufacturing of Suspended and Aligned Nanofibrous Scaffold for Structural Maturation and Synchronous Contraction of HiPSC-Derived Cardiomyocytes
Source: Bioengineering (Basel). 2023 Jun 9;10(6):702. doi: 10.3390/bioengineering10060702 (PMC10295015; doi:10.3390/bioengineering10060702)
Supplement: Supplementary file 1 [file bioengineering-10-00702-s001.zip › Supplymentary Files-bioengineering-2377947/bioengineering-2377947-Supplymentary Information.pdf]

Supplementary Information for

Integrated Manufacturing of Suspended and Aligned Nano-fibrous Scaffold for Structural Maturation and Synchronous Contraction of HiPSC-Derived Cardiomyocytes

Lingling Liu, *et al.*

Corresponding author: Songyue Chen, s.chen@xmu.edu.cn

The PDF file includes:

Figs. S1 to S5

Method- Intracellular Staining for Fluorescence-Activated Cell Sorting (FACS)

Other Supplementary Material for this manuscript includes the following:

Video S1 to S2

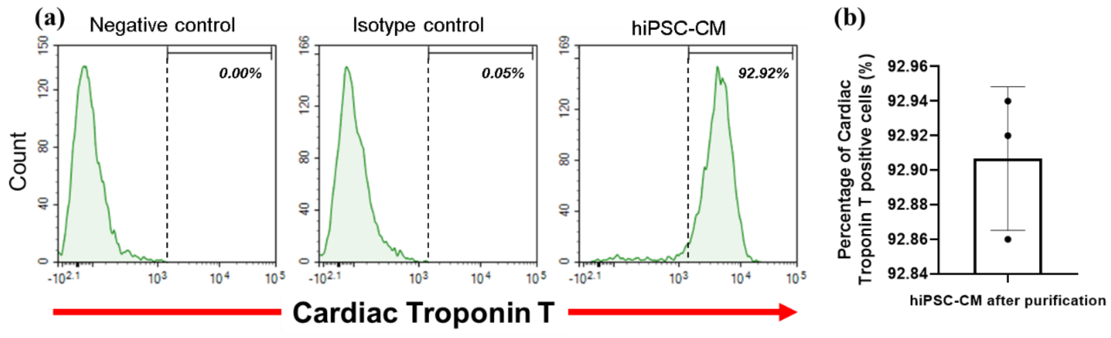

Fig.S1 The purification efficiency of hiPSC-CM in this study. (a) Representative fluorescence-activated cell sorting (FACS) analyses of cardiac Troponin T expression in the human induced pluripotent stem cell-derived cardiomyocytes (hiPSC-CM). In the negative control group, cells without intracellular staining were analyzed by FACS. In the isotype control group, mouse IgG1 was used instead of the primary antibody. (b) The percentage of cardiac Troponin T positive cells in the hiPSC-CM after purification procedures (n=3).

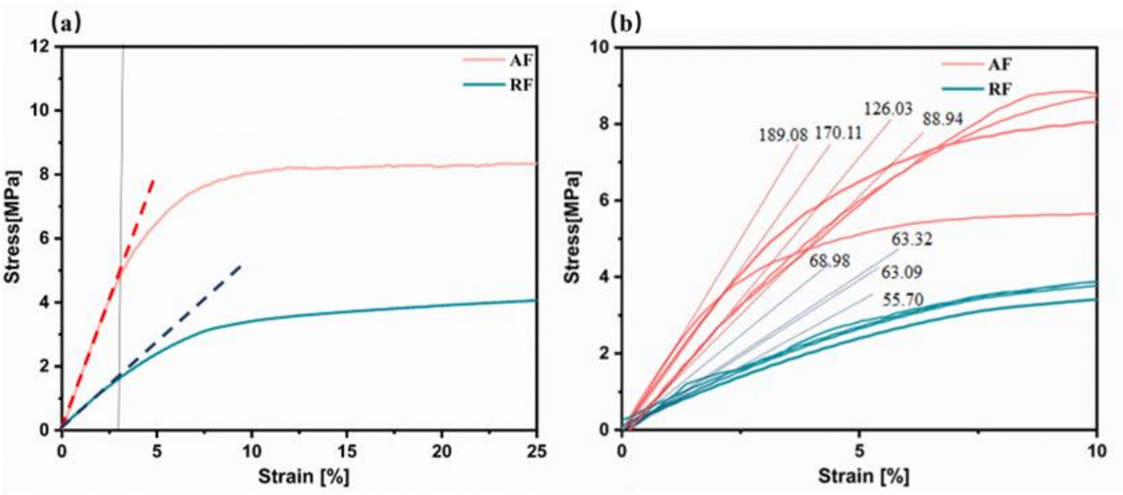

Fig.S2 The stress-strain curve of PCL Aligned Fibers(AF) and Random Fibers(RF)

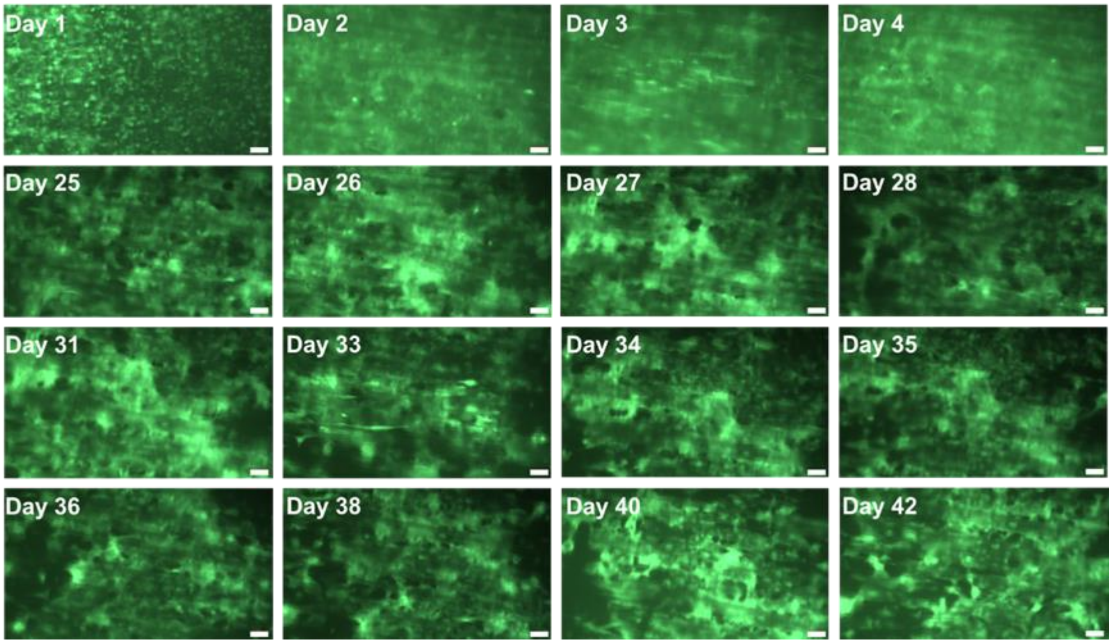

Fig.S3 HiPSC-CMs marked GFP cultured on aligned fibers for 42 days.

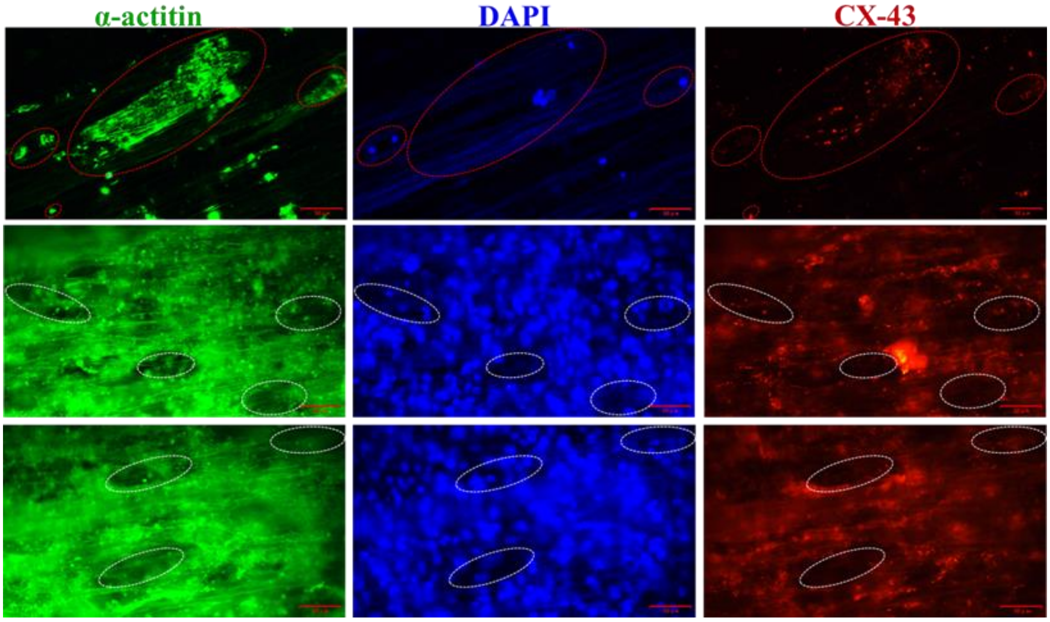

Fig.S4 Immunofluorescence staining of hiPSC-CMs cultured on aligned fibers for 7 days.(Green:α-actinin；Blue: DAPI；Red:CX-43)

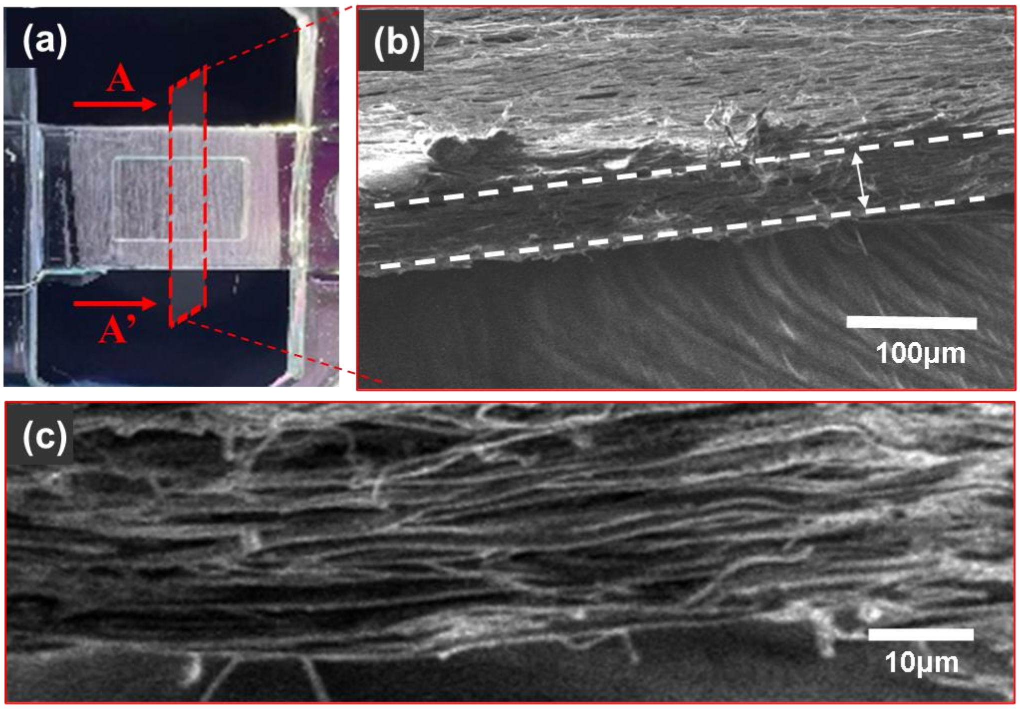

Fig.S5 SEM image of ordered fiber scaffold cross-section.

METHOD

Intracellular Staining for Fluorescence-Activated Cell Sorting (FACS)

All the following steps were performed at room temperature. Cells were dissociated by trypsin and pelleted by centrifugation at 3000 rpm for 5 minutes. Then cells were washed by Intracellular Staining Permeabilization Wash Buffer (BioLegend, 421002) twice, and centrifuged at 3000 rpm for 5 minutes. Cells were fixed with Fixation Buffer (BioLegend, 420801) for 20 min, and blocked in block buffer (10% goat serum in 1×PBS with 0.1%Triton X-100) for 15min. Cells were then incubated for 1 hour in the dark with Cardiac Troponin T monoclonal antibody (used at 1:100, invitrogen, MA5-12960) or normal mouse IgG1 (used at 1:100, santa cruz, sc-3877). Cells were washed twice with wash buffer, then incubated with Alexa Fluor 555 goat anti-mouse IgG secondary antibody (used at 1:500, invitrogen, A32723) in the dark for 1 hour. After twice wash, cells were resuspended in cell staining buffer (Biolend, 420201) and analyzed on a Flow cytometry (NovoCyte D2040R). Data were analyzed by NovoExpress software.
